# Supplementary material for: Neutralization of the Plasmodium-encoded MIF ortholog confers protective immunity against malaria infection
Source: Nat Commun. 2018 Jul 13;9:2714. doi: 10.1038/s41467-018-05041-7 (PMC6045615; doi:10.1038/s41467-018-05041-7)
Supplement: Supplementary file 1 — Supplementary Information [file 41467_2018_5041_MOESM1_ESM.pdf]

Baeza Garcia A *et al.*

Neutralization of the *Plasmodium*-encoded MIF Confers Protective Immunity against Malaria Infection

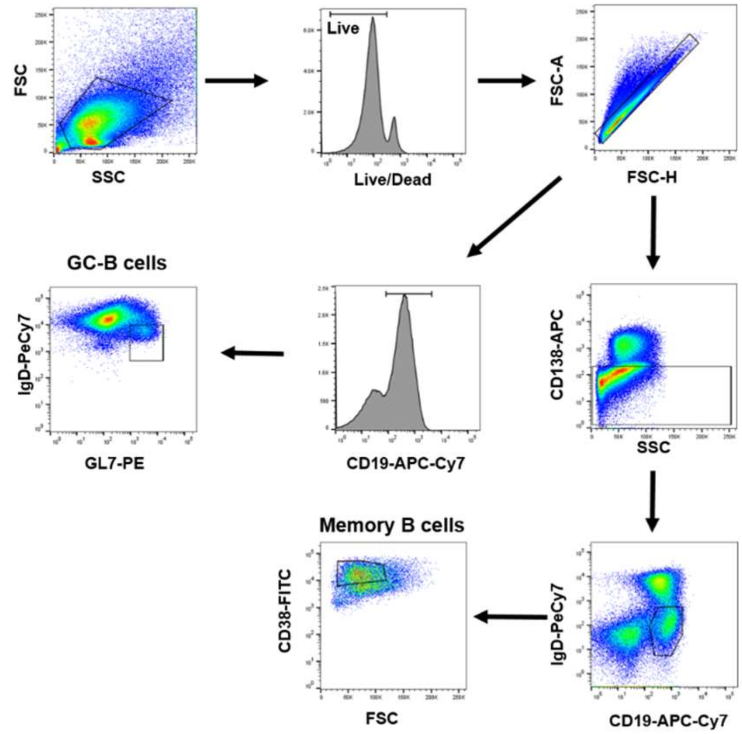

Supplementary Figure 1. Deficient anti-*Plasmodium* antibody response and germinal center formation in the presence of PMIF. Flow cytometry gating strategy used to identify germinal center B cells ($CD19^{+}IgD^{-}GL7^{+}$) and memory B cells ($CD19^{+}CD138^{-}IgD^{-}CD38^{+}$).

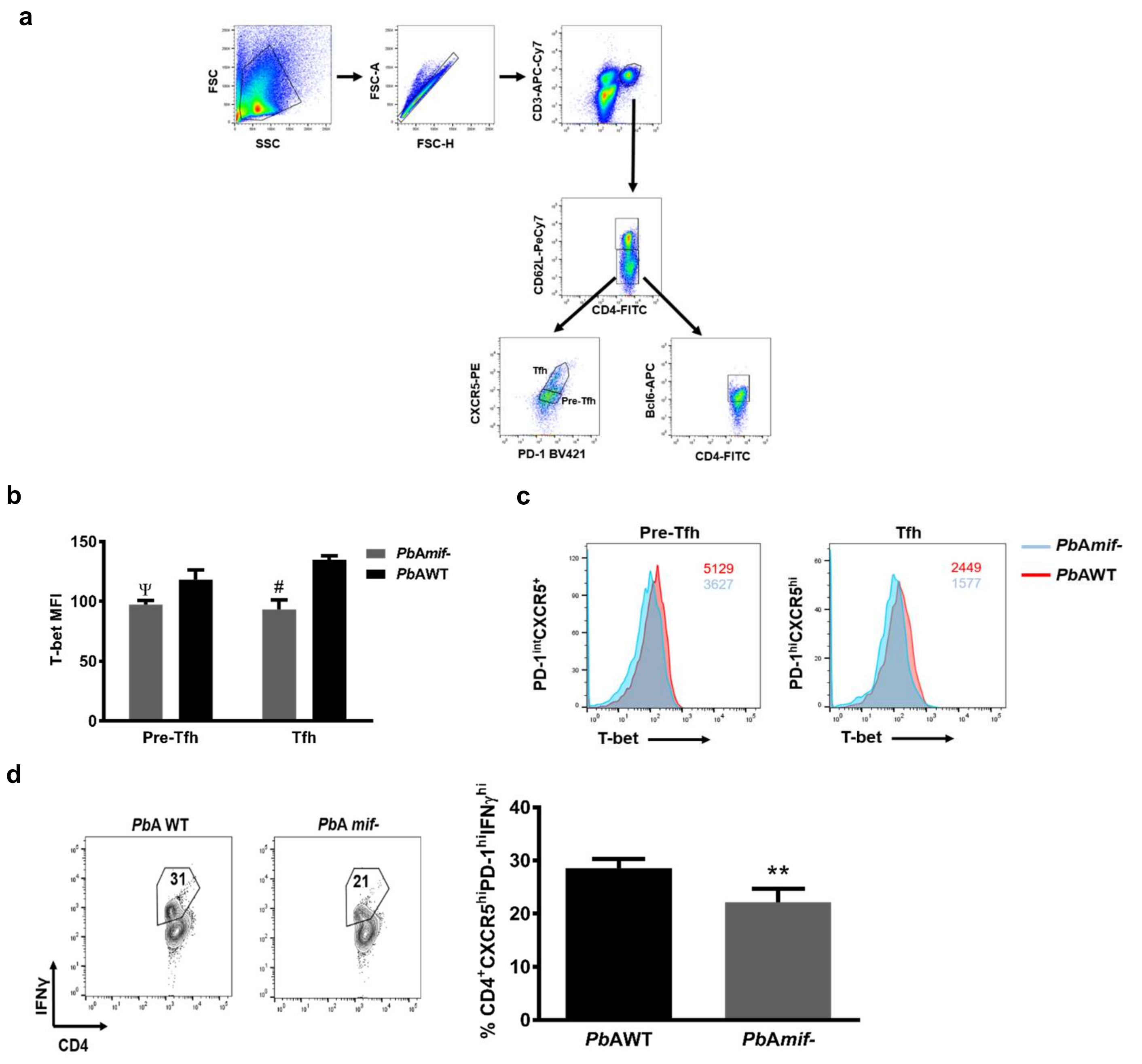

Supplementary Figure 2. PMIF promotes T-bet and IFN-γ expression during Tfh cell differentiation. **a,** Flow cytometry gating strategy used to identify and immunophenotype pre-Tfh cells ($CD4^+CD62L^-CXCR5^{int}PD\text{-}1^{int}$) and Tfh cells ($CD4^+CD62L^-CXCR5^{hi}PD\text{-}1^{hi}$). BALB/cJ mice were infected with 10^6 *Pb*AWT or *Pb*A*mif-* iRBCs. On day 6, splenocytes were isolated and the expression of the transcription factor T-bet, **b** and **c** by pre-Tfh and Tfh cells was measured. Results are from three separate experiments. Bars represent the mean of 12 mice ± SD. Ψ$p<0.001$; #$p<0.0001$ by two-way ANOVA. **d,** Percentage of Tfh cells expressing the pro-inflammatory cytokine IFN-γ. Results are from three separate experiments. Bars represent the mean of 12 mice ± SD; **$p<0.05$ by Mann-Whitney test.

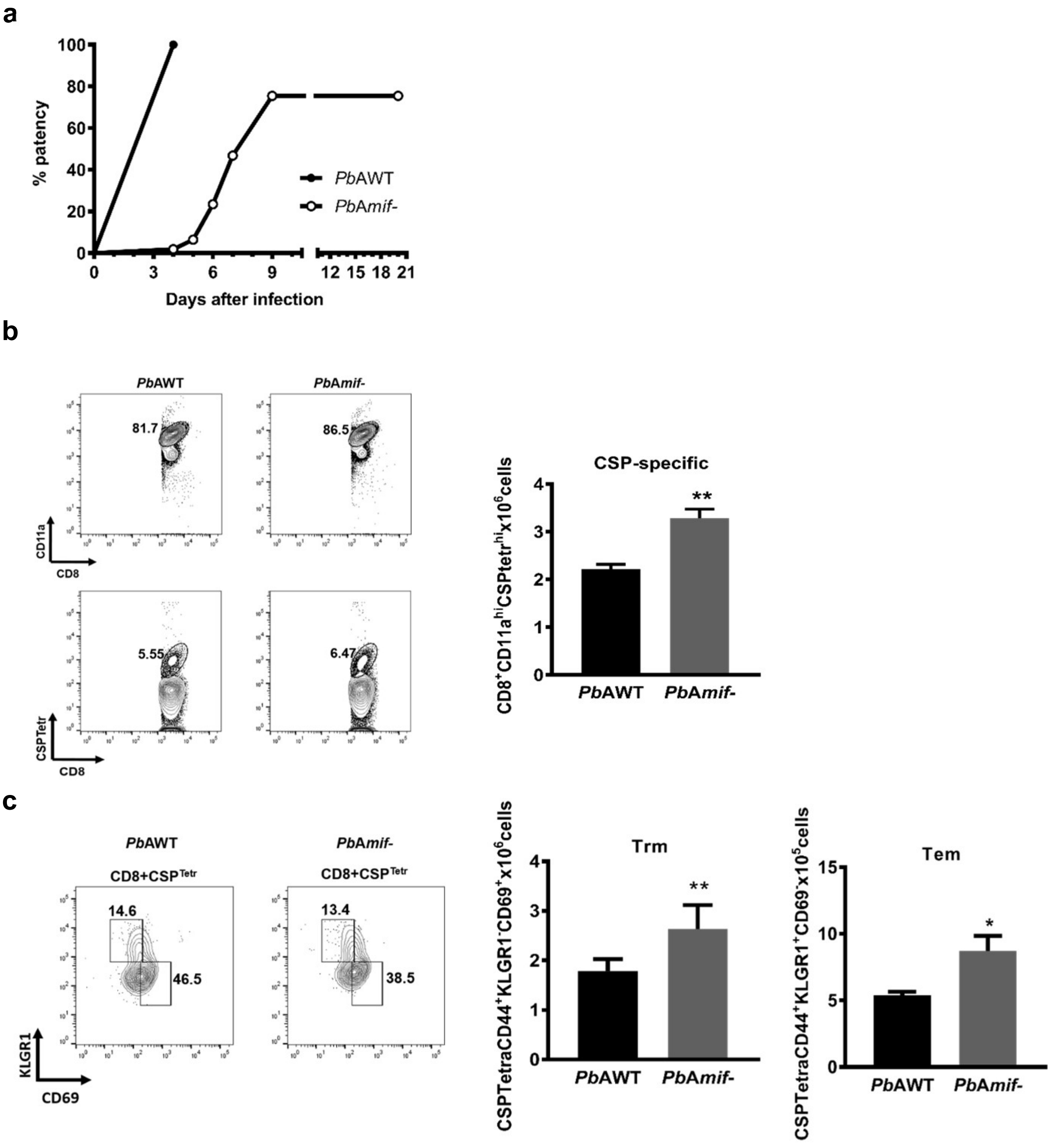

Supplementary Figure 3. PMIF absence increases liver immunity against PbA. **a,** Kaplan-Meyer plots showing the percentage of BALB/cJ mice with blood-stage patency after challenge by i.v. injection with $2x10^3$ *Pb*AWT (●) or *Pb*A*mif-* (○) sporozoites. Patency was determined by microscopic enumeration of blood-stage patency by thin blood smears following day 3 of infection. Data are from two independent experiments, with 10 animals per group; p=0.0001 by Log-rank (Mantel Cox) test. **b,** Representative plots and absolute numbers of CSP-specific CD8 T cells ($CD8^+CD11a^{hi}CSPTetra^{hi}$) in the livers of BALB/cJ mice infected 7 days previously with 2x103 *Pb*AWT or *Pb*A*mif-* sporozoites. Results are from two separate experiments. Bars represent the mean of 6 mice ± SD; **p=0.0079 by Mann-Whitney test. **c,** Representative plots and absolute numbers of CSP-specific tissue resident memory cells (Trm: $CD8^+CD11a^{hi}CSPTetr^{hi}CD44^{hi}KLGR1^-CD69^+$) and effector memory CD8 T cells (Tem: $CD8^+CD11a^{hi}CSPTetr^{hi}CD44^{hi}KLGR1^+CD69^-$) at day 7 after infection. Results are from two separate experiments. Bars represent the mean of six mice ± SD; *p=0.0317, **p=0.0079 by Mann-Whitney test.

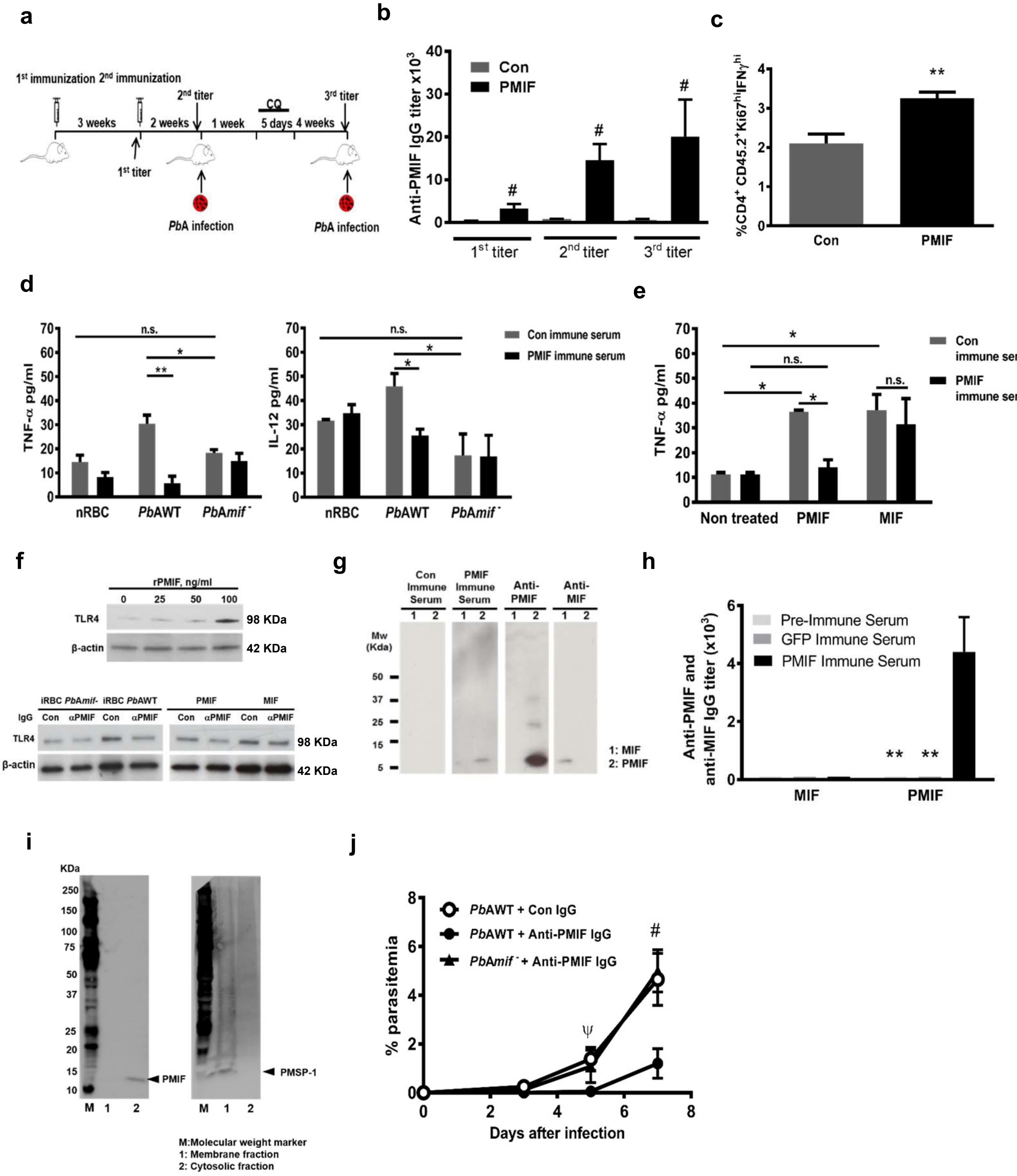

a
1st immunization 2nd immunization
3 weeks
2 weeks
2nd titer
1 week
CQ
5 days
4 weeks
3rd titer
1st titer
PbA infection
PbA infection
b
Anti-PMIF IgG titer x10³
Con
PMIF
1st titer
2nd titer
3rd titer
c
%CD4⁺ CD45.2⁺Ki67hiIFNγhi
Con
PMIF
d
TNF-α pg/ml
IL-12 pg/ml
n.s.
Con immune serum
PMIF immune serum
nRBC
PbAWT
PbAmif⁻
e
TNF-α pg/ml
Con immune serum
PMIF immune serum
Non treated
PMIF
MIF
f
rPMIF, ng/ml
TLR4
β-actin
98 KDa
42 KDa
iRBC PbAmif-
iRBC PbAWT
IgG
Con
αPMIF
g
Con Immune Serum
PMIF Immune Serum
Anti-PMIF
Anti-MIF
Mw (Kda)
1: MIF
2: PMIF
h
Anti-PMIF and anti-MIF IgG titer (x10³)
Pre-Immune Serum
GFP Immune Serum
PMIF Immune Serum
MIF
PMIF
i
KDa
PMIF
PMSP-1
M:Molecular weight marker
1: Membrane fraction
2: Cytosolic fraction
j
PbAWT + Con IgG
PbAWT + Anti-PMIF IgG
PbAmif⁻ + Anti-PMIF IgG
% parasitemia
Days after infection

Supplementary Figure 4. A self-amplifying RNA encoding PMIF elicits specific neutralizing anti-PMIF IgG and PMIF responsive CD4 T cells. a, Experimental design: BALB/cJ mice were immunized at weeks 0 and 3 with a *pmif* or control (Con) RNA replicon and injected i.p. with 10^6 *Pb*AWT iRBCs. A cohort of infected mice was treated with chloroquine (CQ) on days 7–12 to eliminate parasites; mice were re-infected with 10^6 *Pb*AWT iRBCs 4 weeks later. **b,** Anti-PMIF immunoglobulin titers 3 weeks after first immunization (1st titer), 2 weeks after second immunization (2nd titer), and 5 weeks following infection (3rd titer). Results are from two separate experiments. Bars represent the mean of 10-15 mice ±SD; #p<0.0001 by Mann-Whitney test. **c,** Frequency of PMIF responsive CD4 T cells ($Ki67^{hi}CD4^{+}$) expressing IFN-γ in the spleens of *pmif* or control RNA immunized mice isolated 15 days after second immunization and stimulated *ex vivo* with recombinant PMIF in the presence of CD45.1 splenic antigen-presenting cells and Brefeldin A. Data are from two independent experiments. Bars represent the mean of 6 mice ± SD; **p<0.01 between PMIF vs. GFP control by 2-tailed Mann-Whitney test. **d,** TNF-α and IL-12 content in supernatants from murine bone marrow-derived macrophages (10^6 per well) after stimulation for 18 hrs with uninfected RBCs (nRBCs), RBCs infected with *Pb*AWT, or RBCs infected with *Pb*A*mif*- (each at 20 iRBCs per macrophage) in the presence of control (Con) immune serum or PMIF immune serum (2 µl per ml). Data shown are mean ± SD of three independent experiments performed in duplicate; *p<0.05 and **p<0.01 between PMIF vs. Con immune serum by Mann-Whitney test. **e,** TNF-α content in supernatants from bone marrow-derived macrophages (10^6 per well) after stimulation for 18 hrs with PMIF or MIF, each at 100 ng/ml, in the presence of PMIF or Con immune serum (2 µl per ml); *p<0.05 and n.s.: non-significant by Mann-Whitney test. **f,** (*upper*) TLR4 protein production by murine Bone Marrow-Derived Macrophages (BMDM) after stimulation in vitro for 18 hrs with rMIF**.** (*lower*) Impact of anti-PMIF IgG (αPMIF) on TLR4 production in BMDMs induced by treatment with *PbAmif*- or *PbA*WT iRBC lysates (*left panel*) or recombinant PMIF or murine MIF (*right panel*). Lysates or recombinant proteins were incubated with 100 µg/ml of IgG (RT, 1 hr) purified from mice immunized with a control (Con) or PMIF-expressing RNA replicon (αPMIF), added to BMDM for 18 hrs, and the BMDM lysates probed for the presence of TLR4. β-actin was used as a loading control. **g**, Detection of recombinant PMIF or mouse MIF by immunoblotting using serum from mice immunized with replicons encoding control RNA or PMIF RNA. Polyclonal antibodies raised to PMIF (Anti-PMIF) and mouse MIF (Anti-MIF) served as positive controls. *Lane 1*: recombinant mouse MIF, *Lane 2*: recombinant PMIF, both at 100 ng per lane. **h,** Detection of anti-PMIF and anti-MIF immunoglobulin titers in pre-immune, PMIF, and Con immune serum. Titers were determined by ELISA using immobilized recombinant PMIF or mouse MIF and expressed as the mean of anti-PMIF or anti-MIF titers in all studied mice. Data are from two independent experiments. Bars represent the mice of 10 animals ± SD; **p<0.01 by Mann-Whitney test. **i**, Detection of PMIF (*left*) and PMSP-1 (*right*) in merozoites membrane and cytosolic fractions. *Lane 1*: membrane fraction, *Lane 2:* cytosolic fraction. **j**, Control (Con) or Anti-PMIF IgG (200 µl dosed at 1 mg/ml) was administrated i.p. to naïve BALB/cJ mice on days -1, 1, 2, and 3 post-infection with 10^6 PbAWT or PbAmif- iRBCs and parasitemia followed by flow cytometry. Data are from two independent experiments. Bars represent the mean of 8 mice ± SD; Ψ p<0.001; #p<0.0001 by two-way ANOVA.

a

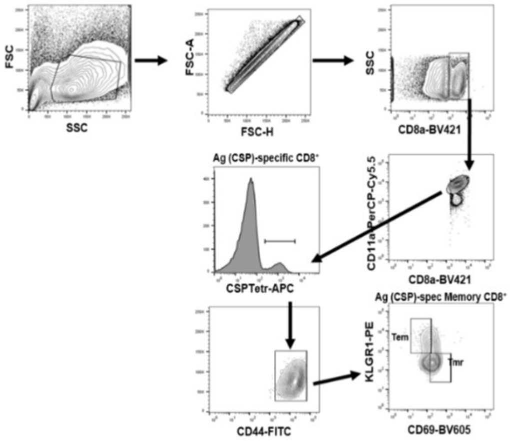
FSC
SSC
FSC-A
FSC-H
SSC
CD8a-BV421
Ag (CSP)-specific CD8+
CSPTetr-APC
CD11a-PerCP-Cy5.5
CD8a-BV421
CD44-FITC
Ag (CSP)-spec Memory CD8+
KLGR1-PE
Tem
Trm
CD69-BV605

b

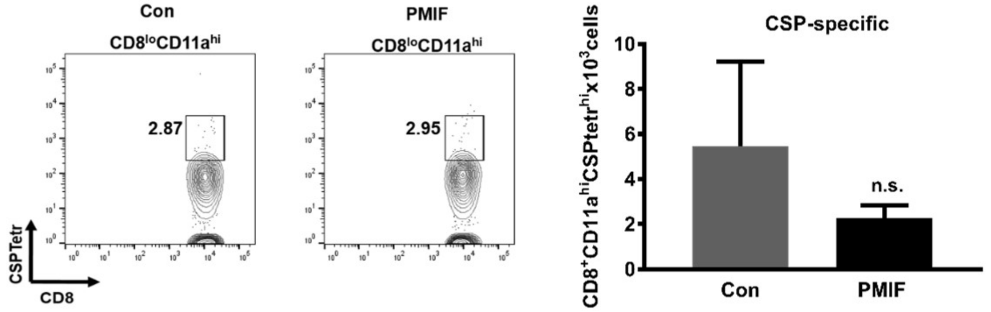
Con
CD8loCD11ahi
2.87
PMIF
CD8loCD11ahi
2.95
CSPTetr
CD8
CSP-specific
CD8+CD11ahiCSPtetrhix10-3cells
n.s.
Con
PMIF

c

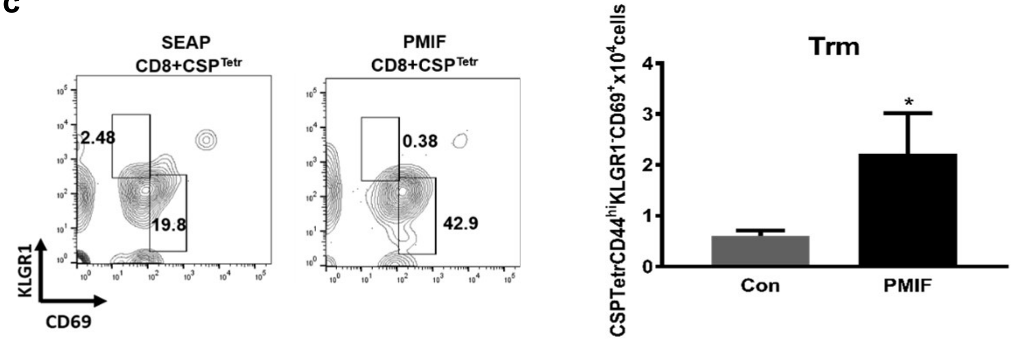
SEAP
CD8+CSPTetr
2.48
19.8
PMIF
CD8+CSPTetr
0.38
42.9
KLGR1
CD69
Trm
CSPTetrCD44hiKLGR1-CD69+x104cells
*
Con
PMIF

d

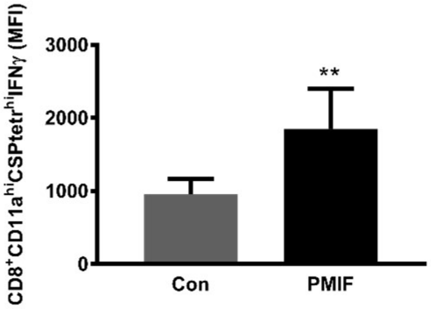
CD8+CD11ahiCSPtetrhiIFNγ (MFI)
**
Con
PMIF

Supplementary Figure 5. PMIF neutralization enhances the development of *Plasmodium* liver memory CD8 T cells. Mice immunized with replicons encoding Con RNA or PMIF RNA were challenged with 2000 *Pb*AWT sporozoites by i.v injection. On day 7 after first or second infection, splenocytes were isolated and the percentage and total number of CD8 T cells measured. **a,** Representative plots and absolute numbers of CSP-specific CD8 T cells ($CD8^{+}CD11a^{hi}CSPTetr^{hi}$) in the spleens of Con and PMIF immunized mice. Results are from two separate experiments. Bars represent the mean of 6 mice ± SD; n.s.= non-significant by Mann-Whitney test. **b,** Representative plots and absolute number of liver CSP-specific tissue resident memory CD8 T cells (Trm: $CSPTetrCD44^{hi}KLGR1^{-}CD69^{+}$) at day 30 after first infection. Results are from two separate experiments. Bars represent the mean of 6 mice ± SD; *p=0.0152 by Mann-Whitney test. **c,** IFNγ expression (MFI) by CD8 Trm cells in the liver of Con and PMIF immunized mice 7 days after first infection with *Pb*AWT sporozoites. Results are from two separate experiments. Bars represent the mean of 6 mice ± SD; **p=0.0087 by Mann-Whitney test.

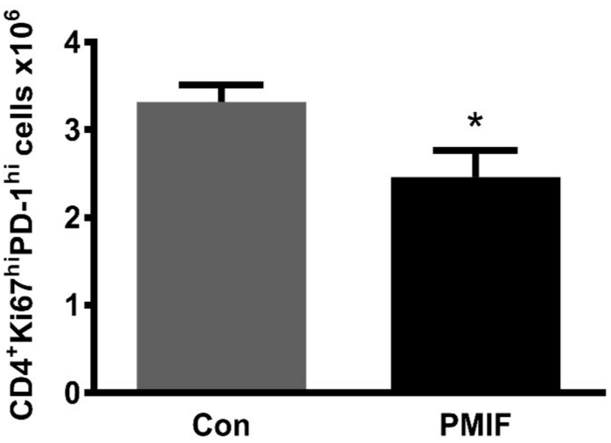

Supplementary Figure 6. Quantitation of *Pb*AWT responsive splenic CD4 T cells expressing PD-1, 7 days after infection in mice immunized with RNA replicons encoding Con RNA or PMIF RNA. Data are representative of two independent experiments. Bars represent the mean of 10 mice ± SD; $*p<0.05$ by Mann-Whitney test.

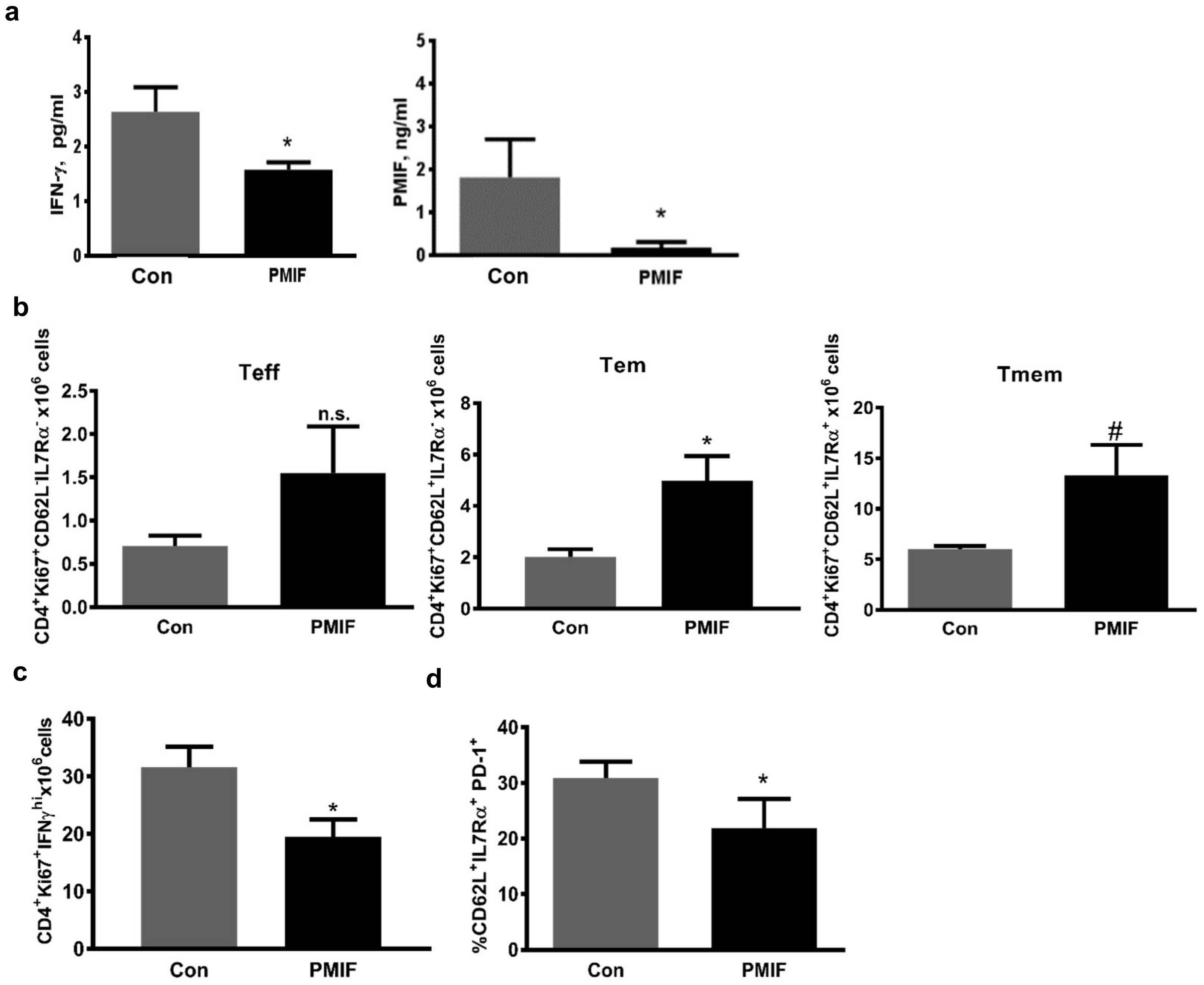

Supplementary Figure 7. Increased of CD4 T cell memory response and decreased T cell exhaustion in PMIF immunized mice. BALB/cJ mice were immunized with replicons encoding Con RNA or PMIF RNA, infected with 10^6 *Pb*AWT iRBCs, and cured by chloroquine treatment 1 week later (*see* Suppl. Fig. 4a). Four weeks later, mice were re-infected with 10^6 *Pb*AWT iRBCs and the splenic CD4 T cells isolated for analysis 7 days later. **a,** Serum levels of IFN-γ and PMIF 7 days after second challenge infection in mice immunized with PMIF versus Con RNA. Data are representative of two independent experiments. Bars represent the mean of 6 mice ± SD; $*p<0.05$ by 2-tailed Mann-Whitney test. **b,** Quantitation of *Pb*AWT responsive CD4 T cell ($Ki67^+CD4^+$) subsets, including T effector (Teff): $CD62L^-IL7R\alpha^-$, T effector memory (Tem): $CD62L^-IL7R\alpha^+$, and T memory (Tmem): $CD62L^+IL7R\alpha^+$. The contribution of each memory CD4 T cell subset is expressed relative to the total number of *Pb*AWT responsive CD4 T cells. **c,** Number of *Pb*AWT responsive CD4 T cells ($CD4^+Ki67^+$) expressing IFN-γ in the spleens of immunized mice. **d,** Frequency of CD4 Tmem ($CD62L^+IL7R\alpha^+$) cells expressing PD-1. Data are representative of two independent experiments. Bars represent the mean of 10 mice ± SD; n.s.: non significant; $*p<0.05$, $\#p<0.0005$ by Mann-Whitney test.

a

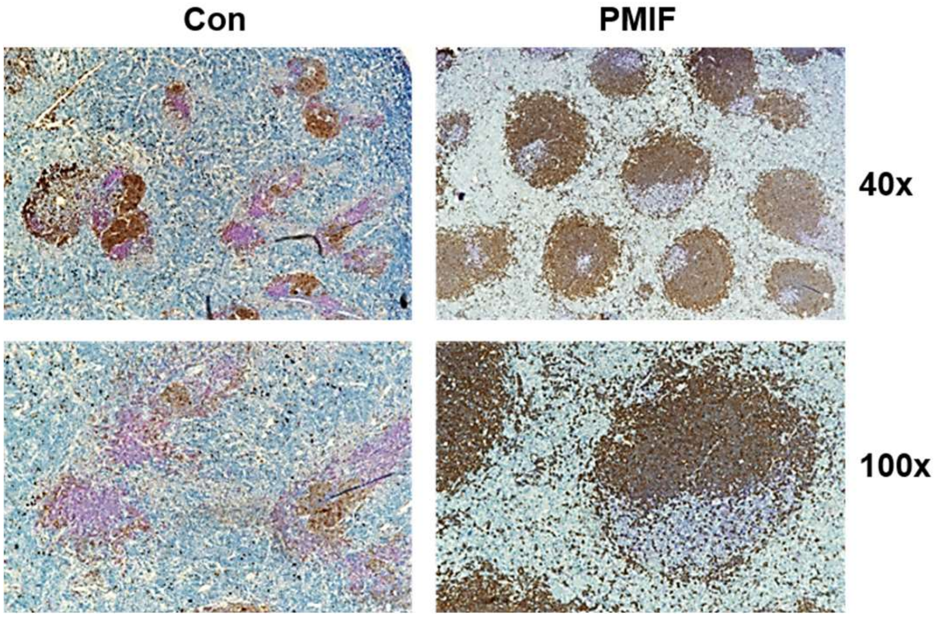

b

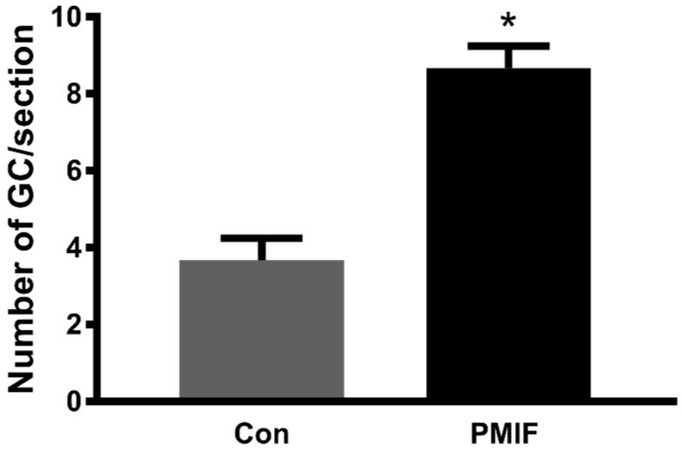

Supplementary Figure 8. Immunohistochemistry of spleens from mice 15 days after infection with 10^6 *Pb*AWT iRBCs and previously immunized with *pmif* RNA or Con RNA replicons. Immunostaining with anti-CD3 (red) and anti-B220 (brown). **a,** Top images for each group show representative regions of spleens (x40) and lower images show a single representative splenic follicle (x100). Data are representative of 4 sections from 2 mice from each experimental group. **b,** Number of germinal centers (GC) in spleens from mice 15 days after infection and previously immunized with PMIF and Con RNA. A GC was enumerated when the limit between the B cell zone (brown) and T cell zone (red) is clearly defined. Data are representative of 3 sections counted from 3 mice per group; mean $\pm$ SD with *$p<0.05$ by 2-tailed Mann-Whitney test.

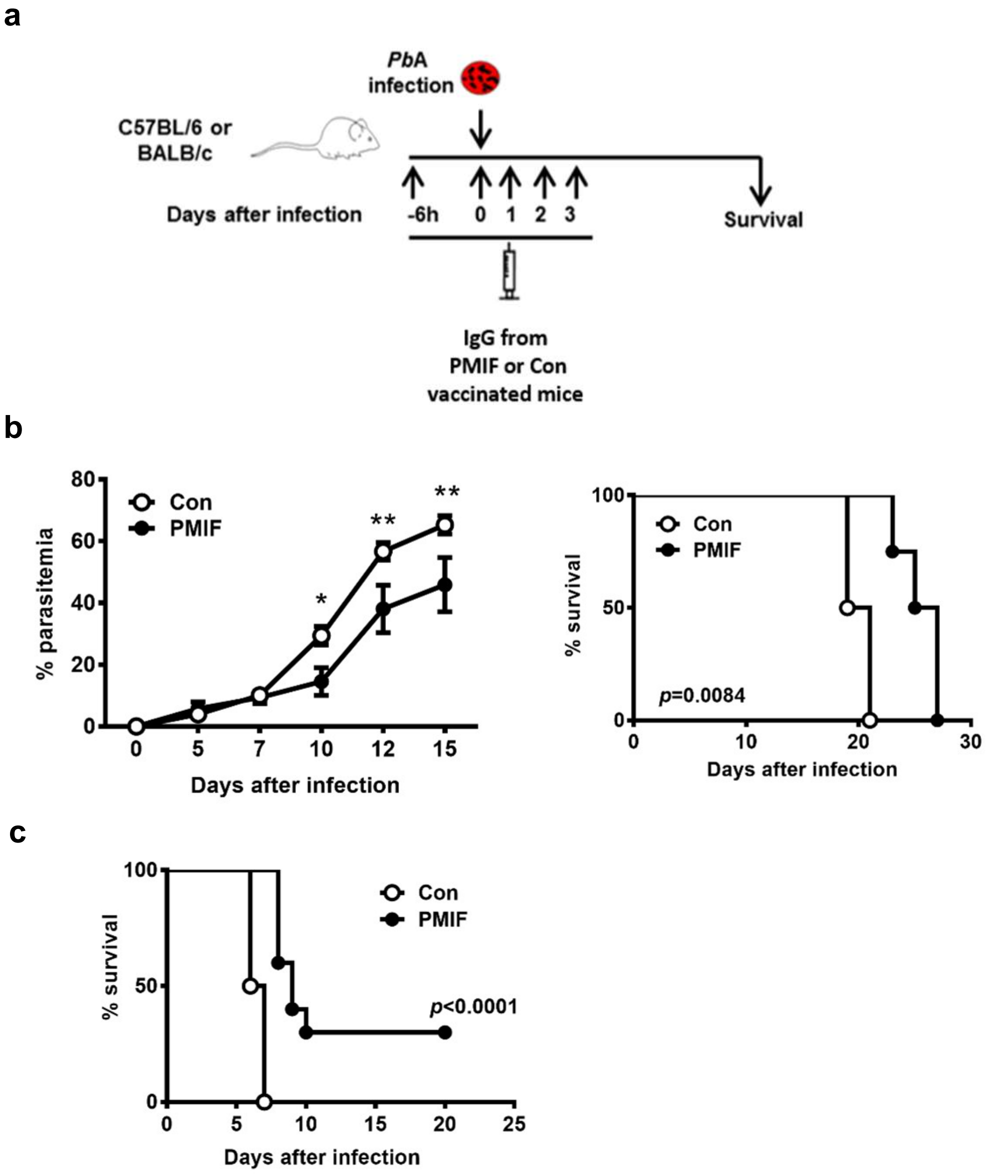

Supplementary Figure 9. **Passive transfer of IgG from PMIF immunized and *Pb*AWT infected mice provides partial protection in both BALB/c and C57BL/6J mice.** **a**, IgG isolated from Control (Con) or PMIF immunized (PMIF) and *Pb*AWT-infected mice was administered i.p. to naïve BALB/c or C57BL/6J mice followed by *Pb*AWT infection. Parasitemia and Kaplan-Meyer survival analysis of *Pb*AWT infected **b**, BALB/c mice, and **c,** C57BL/6 mice. Data are from two independent experiments (5 mice per group) with p values by the Long-rank test (*p<0.05, **p<0.01).

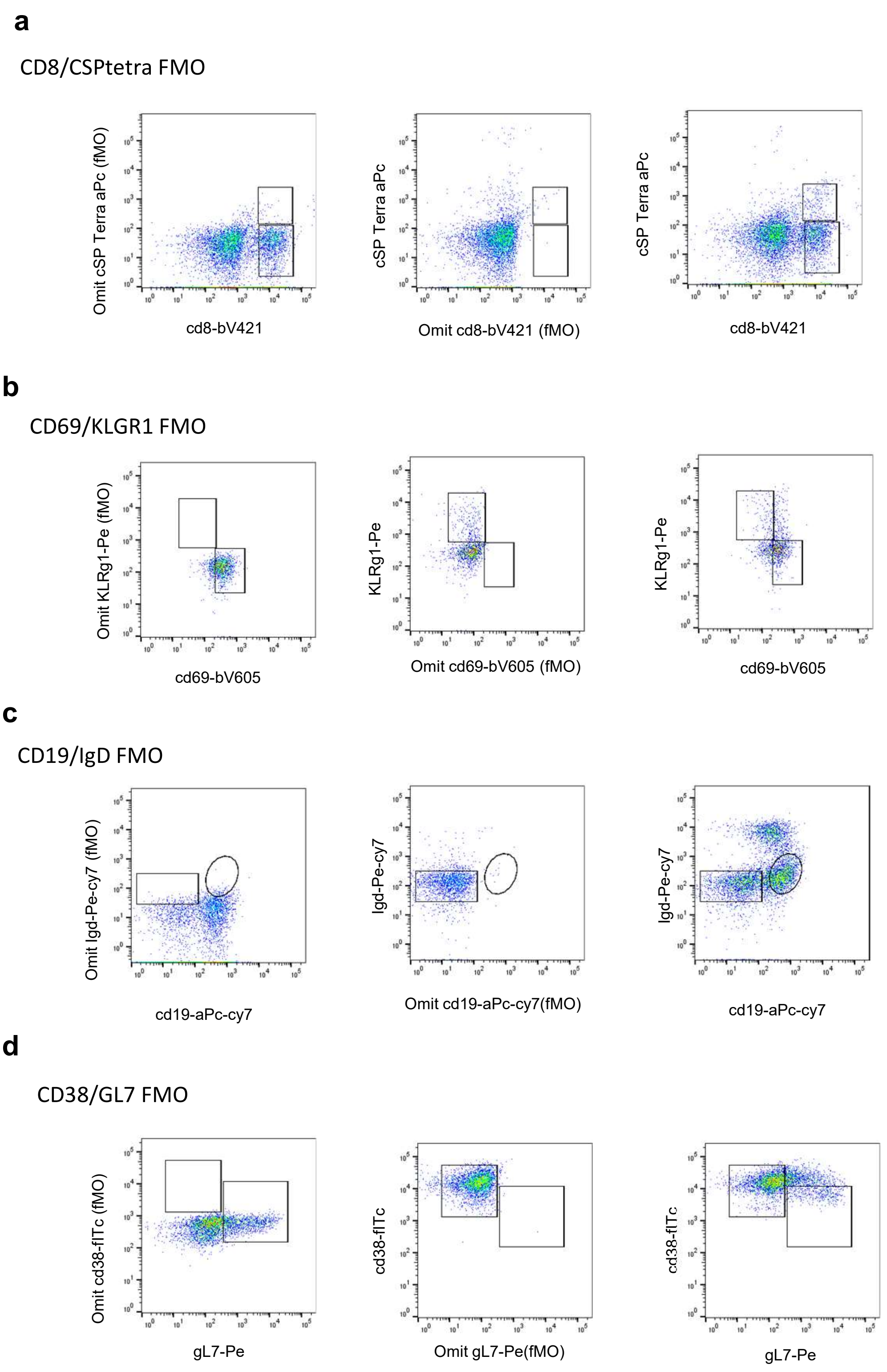

Supplemental Figure 10. Flow cytometry gating strategy. FMO-based gate for **a,** CSP-specific CD8$^+$ T cells, **b,** Trm and Tem CD8$^+$ T cells, **c,** Memory B cells, and **d,** GC B cells.

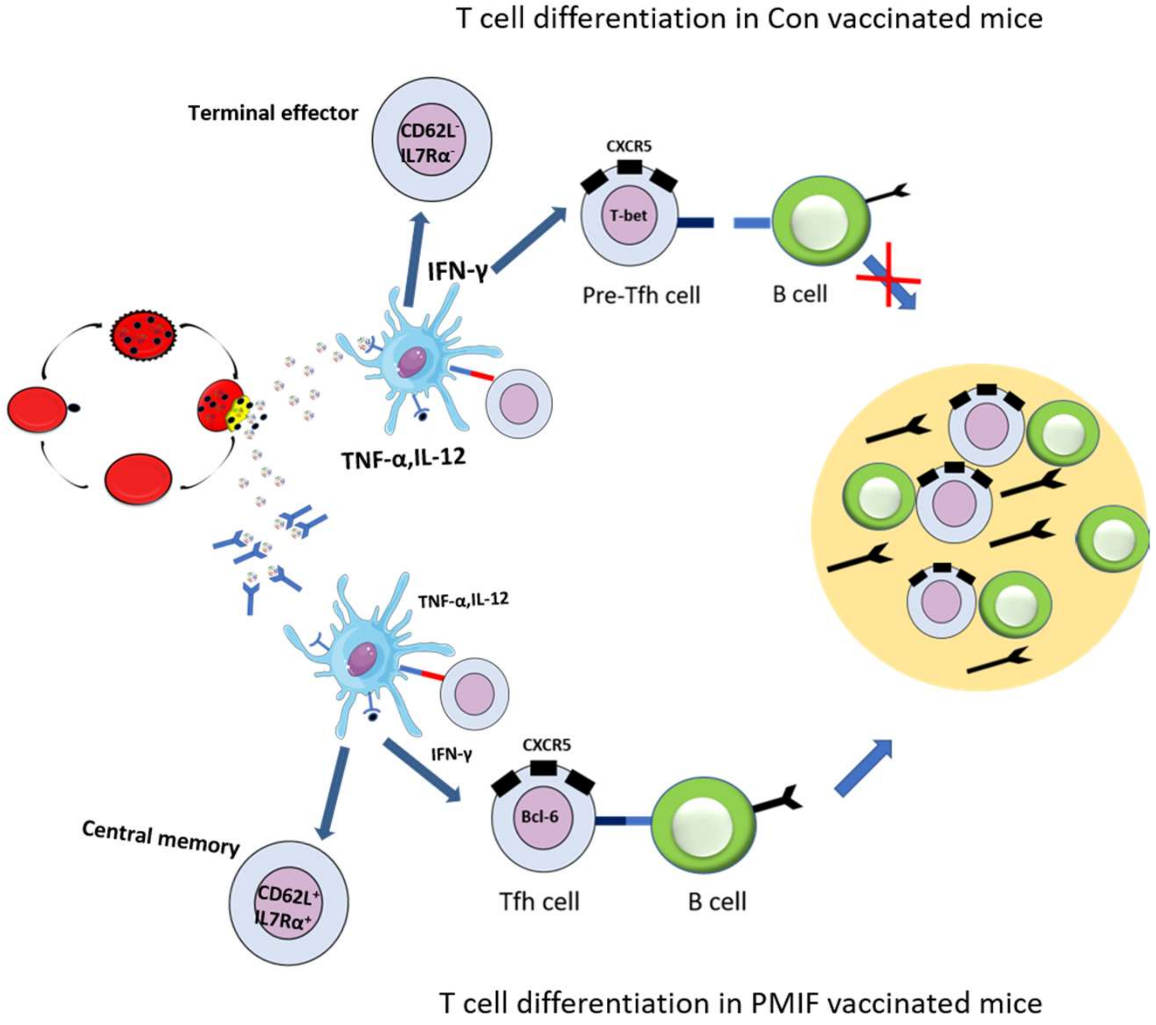

Supplemental Figure 11. Scheme of PMIF action. Anti-PMIF antibodies produced in vaccinated mice block the activity of released PMIF, which reduces myeloid cell activation by limiting the binding between PMIF and the host MIF receptor CD74. Reduced PMIF activity decreases host production of pro-inflammatory cytokines, diminishes Th1 responses, and promotes Tfh responses and the recovery of GC.

a

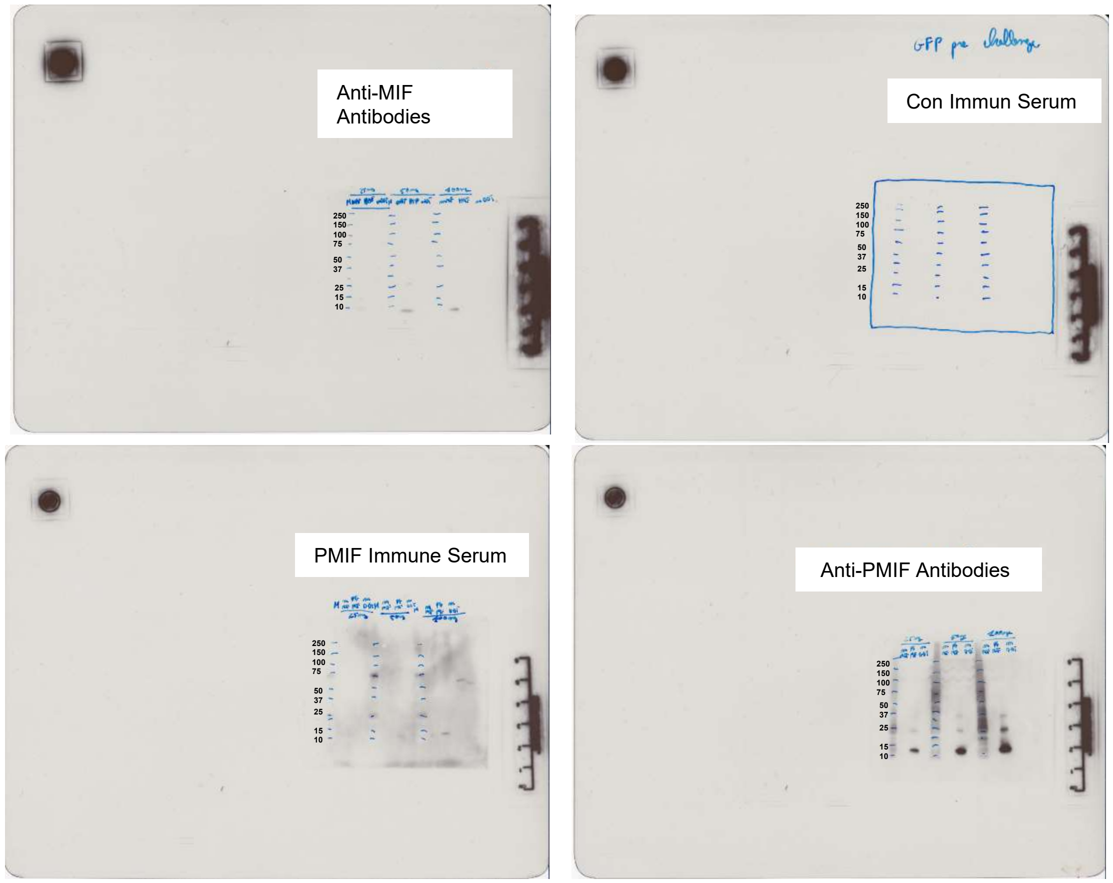
Anti-MIF
Antibodies
Con Immun Serum
PMIF Immune Serum
Anti-PMIF Antibodies
250
150
100
75
50
37
25
15
10

b

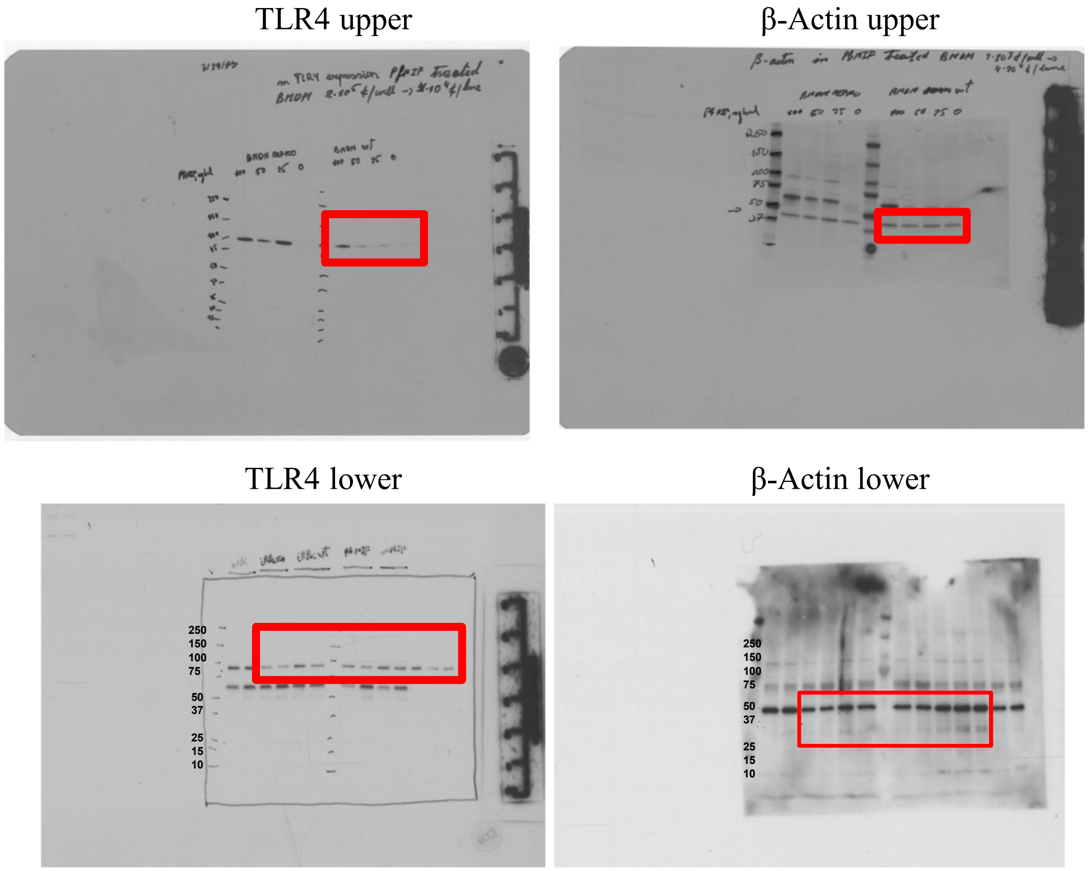
TLR4 upper
β-Actin upper
TLR4 lower
β-Actin lower
250
150
100
75
50
37
25
15
10

c

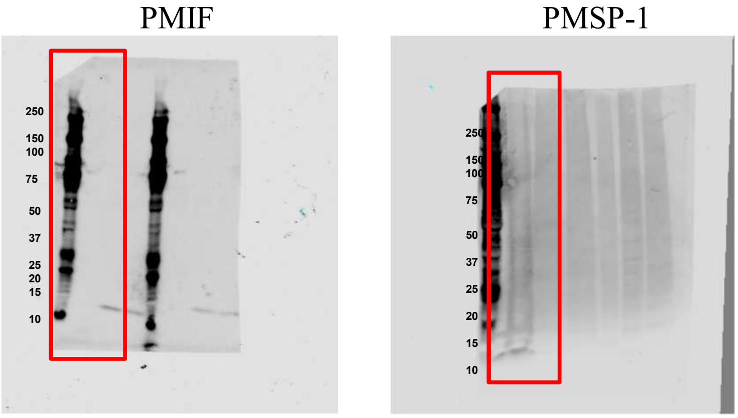

Supplementary Figure 12. Uncropped Scans Figure for Western Blot. a, Supplementary Figure 4f, **b**, Supplementary Figure 4g, **c**, Supplementary Figure 4i.

Target	Flurochrome	Clone	Isotype	Dilution	Provider
CD3	APC-Cy7	17A2	Rat IgG2b, κ	1/500	Biolegend
CD4	FITC	RM4.4	Rat IgG2b, κ	1/500	Biolegend
CD45.1	APC	A20	Mouse IgG2a, κ	1/1000	Biolegend
IL-7Ra	Alexa 405	A7R34	Rat IgG2b, κ	1/500	eBioscience
CD62L	PE-Cy7	MEL-14	Rat IgG2b, κ	1/1200	Biolegend
Ki67	FITC	35	Mouse IgG_1	1/200	BD
T-bet	PerCP-Cy5.5	4B10	Mouse IgG1, κ	1/100	Biolegend
Bcl6	APC	7D1	Rat IgG2b, κ	1/100	Biolegend
IFN-γ	PE/PE-Cy7	XMG1.2	Rat IgG1, κ	1/100	Biolegend
CD19	APC-Cy7	6D5	Rat IgG2a, κ	1/500	Biolegend
CD138	APC	281-2	Rat IgG2a, κ	1/500	Biolegend
IgD	PE-Cy7	11-26c.2a	Rat IgG2a, κ	1/250	Biolegend
GL7	PE	GL7	Rat IgM, κ	1/250	Biolegend
CD38	FITC	90	Rat IgG2a, κ	1/500	eBioscience
CD8	BV421	53-6.7	Rat IgG2a, κ	1/500	Biolegend
CD69	BV605	H1.2F3	Arme Hams IgG	1/500	Biolegend
KLRG1	PE	2F1/ KLRG1	Syri Hams IgG	1/500	Biolegend
CD44	FITC	IM7	Rat IgG2b, κ	1/1000	Biolegend
CXCR5	PE	L138D7	Rat IgG2b, κ	1/500	Biolegend
PD-1	BV421	29F.1A12	Rat IgG2a, κ	1/250	Biolegend

Supplementary Table 1. Antibodies for Cytometry
